# Draft Genome Sequence of *Streptomyces* sp. TP-A0871, a Producer of Heronamide C

**DOI:** 10.1128/genomeA.01429-15

**Published:** 2015-12-10

**Authors:** Hisayuki Komaki, Natsuko Ichikawa, Akira Hosoyama, Nobuyuki Fujita, Yasuhiro Igarashi

**Affiliations:** a Biological Resource Center, National Institute of Technology and Evaluation (NBRC), Chiba, Japan; b NBRC, Tokyo, Japan; c Biotechnology Research Center and Department of Biotechnology, Toyama Prefectural University, Toyama, Japan

## Abstract

*Streptomyces* sp. TP-A0871 produces the polyene macrolactam heronamide C. Here, we report its draft genome sequence to get insight into heronamide biosynthesis and genome-mining for novel secondary metabolites of polyketide and nonribosomal peptide classes. The genome encodes over nine orphan gene clusters for polyketide and/or nonribosomal peptide syntheses.

## GENOME ANNOUNCEMENT

In our screening for inhibitors of tumor cell invasion from microbial secondary metabolites, *Streptomyces* sp. TP-A0871, isolated from soil in Toyama, Japan, was found to produce the polyene macrolactam heronamide C (1). In the present study, we conducted the whole-genome shotgun sequencing of this strain to identify the biosynthetic gene cluster of heronamide and to investigate polyketide synthase (PKS) and nonribosomal peptide synthetase (NRPS) gene clusters.

*Streptomyces* sp. TP-A0871 was deposited in the NBRC culture collection (NBRC 110028). The whole genome of the *Streptomyces* sp. TP-A0871 monoisolate was read by using a combined strategy of shotgun sequencing with GS FLX+ (Roche) (98-Mb sequences, 12-fold coverage) and pair-end sequencing with HiSeq1000 (Illumina) (723 Mb, 89-fold coverage). These reads were assembled using Newbler version 2.6 software, and were subsequently finished using GenoFinisher software (2), which led to a final assembly of 96 scaffold sequences of >500 bp each. The total size of the assembly was 8,921,410 bp, with a G+C content of 71.5%. Coding sequences were predicted by Prodigal (3). PKS and NRPS gene clusters were surveyed as previously described (4).

The genome contains at least four type I PKS, one type II PKS, eight NRPS, and one PKS/NRPS hybrid gene clusters. Among them, three type I PKS gene clusters were divided into several scaffolds, one of which is encoded in scaffolds 35, 39, 54, 57, 65, 66, and 6 (ORF1) but can be assigned to heronamide biosynthetic genes because the PKSs in this cluster show high sequence similarities to those for BE-14106 (5), a macrolactam structurally similar to heronamides. The remaining two PKS clusters show high sequence similarities to the *nig* (6) and *ole* (7) clusters, respectively. A type I PKS gene cluster present in scaffold 6 (ORF67 to ORF69), which was completely sequenced, harbors only two modules, and does not show high sequence similarity to known gene clusters. A type II PKS gene cluster in scaffold 3 is likely for spore pigment production. Eight orphan NRPS gene clusters are present in scaffolds 5, 6, 11, 12, 13, 14, 18, and 25. Based on module numbers in each NRPS cluster, peptide products are speculated to consist of ten, three, three, three, one, two, eight, and six amino-acid residues, respectively. A similarity search suggested that the product of the NRPS in scaffold 11 is a kind of siderophores. A PKS/NRPS hybrid gene cluster present in scaffold 25 encodes four NRPS modules and one PKS module, suggesting that the product comprises four amino acids and one polyketide unit.

During this study, the biosynthetic gene cluster for heronamide F in *Streptomyces* sp. SCSIO 03032 was reported (8), but the sequence data are not available at present. The genome sequence of *Streptomyces* sp. TP-A0871 will provide valuable information to study heronamide biosynthesis and to search novel polyketides and nonribosomal peptides.

### Nucleotide sequence accession numbers.

The draft genome sequence of *Streptomyces* sp. TP-A0871 has been deposited in the DDBJ/ENA/GenBank database under the accession number BBUZ00000000. The version described in this paper is the first version, BBUZ01000000.
